# Distinctively variable sequence-based nuclear DNA markers for multilocus phylogeography of the soybean- and rice-infecting fungal pathogen *Rhizoctonia solani* AG-1 IA

**DOI:** 10.1590/S1415-47572009005000063

**Published:** 2009-12-01

**Authors:** Maisa B. Ciampi, Liane Rosewich Gale, Eliana G. de Macedo Lemos, Paulo C. Ceresini

**Affiliations:** 1 Departamento de Tecnologia, Faculdade de Ciências Agrárias e Veterinárias, Universidade Estadual Paulista, Campus de Jaboticabal, Jaboticabal, SPBrazil; 2 Department of Plant Pathology, University of Minnesota, St. Paul, MNUSA; 3 Departamento de Fitossanidade, Engenharia Rural e Solos, Universidade Estadual Paulista, Campus de Ilha Solteira, Ilha Solteira, SPBrazil; 4 Swiss Federal of Technology Zurich, Institute of Integrative Biology, Plant Pathology, ZurichSwitzerland

**Keywords:** multilocus genotyping, polymorphisms, allelic discrimination, primer design

## Abstract

A series of multilocus sequence-based nuclear DNA markers was developed to infer the phylogeographical history of the Basidiomycetous fungal pathogen *Rhizoctonia solani* AG-1 IA infecting rice and soybean worldwide. The strategy was based on sequencing of cloned genomic DNA fragments (previously used as RFLP probes) and subsequent screening of fungal isolates to detect single nucleotide polymorphisms (SNPs). Ten primer pairs were designed based on these sequences, which resulted in PCR amplification of 200-320 bp size products and polymorphic sequences in all markers analyzed. By direct sequencing we identified both homokaryon and heterokaryon (*i.e.* dikaryon) isolates at each marker. Cloning the PCR products effectively estimated the allelic phase from heterokaryotic isolates. Information content varied among markers from 0.5 to 5.9 mutations per 100 bp. Thus, the former RFLP codominant probes were successfully converted into six distinctively variable sequence-based nuclear DNA markers. Rather than discarding low polymorphism loci, the combination of these distinctively variable anonymous nuclear markers would constitute an asset for the unbiased estimate of the phylogeographical parameters such as population sizes and divergent times, providing a more reliable species history that shaped the current population structure of *R. solani* AG-1 IA.

Classical analyses of the distribution of genetic diversity within and among populations have been used to identify patterns of migration and to reveal cryptic recombination in *Rhizoctonia solani* (Rosewich *et al.*, 1999; Ceresini *et al.*, 2002, 2007; Ciampi *et al.*, 2005, 2008; Linde *et al.*, 2005; Bernardes de Assis *et al.*, 2008), but information on global phylogeography does not exist for any *Rhizoctonia* pathosystem. Phylogeography is the study of historical processes responsible for the contemporary geographic distributions of individuals. Past events that can be inferred include population expansion, population bottlenecks, vicariance and migration (Karl and Avise, 1993; Avise, 2000).

The classical studies on population genetics have used molecular markers, such as RAPD, ISSR, RFLP, and more recently microsatellite loci. However, these molecular markers are not fully suitable for studying the phylogeography of the fungus. A suitable marker would enable the implementation of the genealogical approach and the application of coalescent and phylogenetic tools for population-level questions (Brito and Edwards, 2008). Sequence variation from several stretches of anonymous DNA regions have been suggested as the marker of choice to infer phylogeographical history of species, for containing multiple and linked single nucleotide polymorphisms (SNPs), essential for constructing gene genealogies (Karl and Avise, 1993; Brito and Edwards, 2008). SNPs have simple patterns of variation, the potential for automated detection, low mutation rates (about 10^-8^ to10^-9^), and thus, low levels of homoplasy (Brito and Edwards, 2008). In addition, many more tests for elucidating population parameters and historical demography (*e.g.*, calculating deviations from neutrality, population size changes, divergence times, and recombination) exist for data derived from sequence-based markers than for any other marker (Brumfield *et al.*, 2003). With the costs of high throughput sequencing constantly getting reduced, analysis of nuclear DNA sequence variation is becoming more convenient and appropriate for phylogeography, population genetics, and phylogenetic studies (Zhang and Hewitt, 2003; Hayashi *et al.*, 2004).

The aim of this study was to develop a series of anonymous nuclear DNA sequence-based markers suitable for studies of phylogeography of the rice- and soybean infecting fungus *R. solani* AG-1 IA, based on original RFLP loci (Rosewich *et al.*, 1999), to detect multiple SNPs. Our hypothesis was that these anonymous nuclear markers are distinctively variable, and their combination would constitute an asset for the unbiased estimate of the phylogeographical parameters such as population sizes and divergence times.

We sampled 14 soybean-infecting *R. solani* AG-1 IA isolates (Table 1), from which anastomosis grouping and pathogenicity was determined previously (Fenille, 2001; Meyer, 2002; Costa-Souza *et al.*, 2007). These isolates represent distinct ITS-5.8S rDNA haplotypes detected in Brazil (Ciampi *et al.*, 2005). We developed seven sequencing markers based on seven pUC18 cloning vectors containing genomic DNA fragments previously used as RFLP probes (Rosewich *et al.*, 1999) and considered suitable to genotype *R. solani* AG-1 IA populations in the United States since they were polymorphic and also allowed allelic discrimination in heterokaryons (Rosewich *et al.*, 1999). Plasmids containing the fungal genomic sequences were sequenced with M13 vector primers. Chromatograms were assembled by SEQUENCHER v. 4.6 (Gene Codes Corporation) and a consensus sequence for each probe was computed from both forward and reverse sequences. Based on the consensus sequences, ten primer pairs were designed (ranging from 20 to 22 bp, Table 2) to amplify each specific locus, to further sequence multiple loci and to screen isolates for SNPs at each locus. Using the PRIMER3 RELEASE 1.0 software (Rozen and Skaletsky, 2000), all primers were projected to generate PCR products of 200-320 bp. Primers named “L” were projected to amplify a fragment from the 5'-end of a respective clone sequence and primers named “R” to amplify a fragment from the 3'-end.

A preliminary study to assess the new primers' efficacy in amplification by PCR was carried out by using a sub-sample of three soybean-infecting *R. solani* AG-1 IA isolates (SJ13, SJ19, and SJ36) and one rice-infecting isolate (3F6) (Table 1). Each primer pair was also tested on the original plasmid clone. PCR amplifications were performed separately for each locus in a 20 μL final volume. The reaction mixture contained 5 to 15 ng genomic DNA, 2 μL 10x PCR buffer, 0.4 mM dNTPs mixture, 10 pmol of each specific primer pair, and 1 U of *Taq* polymerase. The initial denaturation step was done at 96 °C for 2 min, followed by 35 cycles of 96 °C for 1 min 60 °C for 1 min and 72 °C for 1 min, with a final elongation step at 72 °C for 5 min. The amplicons were then sequenced and surveyed for SNPs among the four isolates. Markers with adequate amplification efficacy for all four initial isolates were selected to amplify all 18 fungal isolates listed in Table 1, using the PCR conditions described above. In this manner, a set of markers for genotyping *R. solani* AG-1 IA isolates was developed by multiple loci-sequencing.

To separate distinct alleles within heterokaryons, PCR products showing one or more double peaks in both sequencing directions were cloned into a plasmid vector using the TOPO TA^®^ cloning kit (Invitrogen). For each locus, eight clones were recovered per isolate. Plasmidial DNA was extracted following a standard protocol (Sambrook *et al.*, 1989), amplified and sequenced using M13 primers. Chromatograms were assembled and analyzed by SEQUENCHER v. 4.6 program (Gene Codes Corporation), generating consensus sequences in FASTA format.

We searched for homolog sequences at NCBI GenBank (Benson *et al.*, 2007), using BLASTn and BLASTx (Altschul *et al.*, 1997). The sequences of each locus were aligned by using the CLUSTALX program (Thompson *et al.*, 1997). SNPs identification and characterization was performed by means of the CLOURE program (Kohli and Bachhawat, 2003), accentuating only distinct nucleotides related to the first sequence of the alignment. Identification of haplotypes (and isolates who shared it), as well as the number and position of polymorphic sites was done by the SNAP WORKBENCH program (Price and Carbone, 2005). Haplotype diversity (Hd) measures and respective sample standard deviations were calculated according to Nei (1987). Nucleotide diversity or the average number of differences per site between two homologous sequences (π) was also calculated according to Nei (1987). For each marker, π values were estimated as an average among all comparisons. The average number of nucleotide differences among sequences was calculated according to Tajima (1993). All measures were estimated using the program DNASP v. 4.5 (Rozas *et al.*, 2003).

The consensus sequences of seven *R. solani* AG-1 IA clones (probes) from Rosewich *et al.* (1999) exhibited sizes ranging 543-1023 bp and their respective GenBank accession numbers are EU907366-EU907372. Comparisons between these sequences and DNA sequences from NCBI GenBank did not result in any significant matches using BLASTn tool (Altschul *et al.*, 1997). However, BLASTx (Altschul *et al.*, 1997) comparisons resulted in partial identity of most probe sequences with protein coding sequences of basidiomycetes, such as *Laccaria bicolor*, *Coprinopsis cinerea,Cryptococcus neoformans*, and of some ascomycetes, such as *Pichia guilliermondii* and *Phaeosphaeria nodorum.* Only probe R68 showed no similarity with any sequence from GenBank (comparisons done in 2009-02-08).

From combinations preliminarily tested on both four *R. solani* AG-1 IA isolates and the respective clones, eight primer pairs resulted in PCR products. Even though successful PCR amplification was obtained for markers R61L, R78L, R111R, and R116R using the initial sample of four isolates, positive amplifications were not obtained when the fungal isolates sample was increased to 18 isolates. Probably for these loci, a new set of primers should be designed. We identified SNPs in all loci surveyed, with polymorphism levels varying from one to 18 polymorphic sites. The highest number of SNPs was detected for marker R68L, with 18 mutations along 303 bp, while 0.5 to 4.2 mutations per 100 bp were detected in the other markers (Table 3). This locus showed either the highest nucleotide diversity level on polymorphic sites (π = 0.25), while this value ranged 0.003-0.013 for other markers, or the highest average number of nucleotide differences (*k* = 7.686; *k* ranging 0.545-4.176 for others). Haplotype diversity (Hd) measures were very different among markers, and varied from 0.55 (for R148R locus) to 0.94 (for R44L locus). Nucleotide diversity levels or the average number of differences per site between two homologous sequences (π) varied from 0.003 for locus R148R to 0.013 for locus R116L. The average number of nucleotide differences (k) among analyzed sequences was also markedly distinct, showing the lowest value of 0.55 for locus R148R and the highest one of 7.69 for locus R68L, which was the most polymorphic locus among the nuclear markers developed.

Except for two loci (R44L and R68L), indels were found in all loci. Heterokaryotic isolates were detected for most loci, varying from 20% (R116L) to 88% (R44L) of the total isolates; the only exception was locus R148R, for which none heterokaryotic isolate was detect. Cloning and sequencing PCR products showed to be efficacious in resolving DNA bases ambiguity from alleles composing the heterokaryons. We sequenced eight clones of each isolate for each marker, and this strategy seemed to be sufficient for covering the whole allelic variation present in the fungal sample tested. According to the variation detected in isolates of this preliminary sub-sample, six markers were selected for sequencing the total sample of *R. solani* AG-1 IA isolates, listed in Table 1. A general description of marker variation is presented in Table 3, and additional information, such as haplotype frequency, and identification of isolates sharing each haplotype, as well as polymorphic positions within the sequences are presented in a supplementary file (Table S1, available as online content).

Thus, the seven codominant RFLP probes were successfully converted into six distinctively variable sequence-based nuclear DNA markers. The application of the BLASTx tool from NCBI resulted in the detection of only partial DNA base identity of the sequences from the seven RFLP probes with protein coding sequences from few basidiomycete species. The low levels of identity (reflected by similarity with only very short fragments of such protein coding genes) suggest that these sequences of nuclear DNA fragments constitute uncharacterized anonymous regions, probably associated with non-coding regions of the *R. solani* AG-1 IA genome. Up to now, only five complete genomes of basidiomycetes are available: *Coprinopsis cinerea* (accession number NW_001885114), *Phanerochaete chrysosporium* (AADS00000000), *Cryptococcus neoformans* (AAEY00000000), *Ustilago maydis* (AACP00000000), and *Laccaria bicolor* (ABFE01000000). The scarce genomic information for basidiomycetes in general and the current lack of public information from any *Rhizoctonia* genome would explain the low similarity found among the sequences from these *R. solani* AG-1 IA probes and genes characterized until now. In fact, the first genome of a *R. solani* anastomosis group (the potato-infecting AG-3) has been completed in 2008 by the J. Craig Venter Institute and North Carolina State University (funded by US Department of Agriculture) but it is not yet publicly available for comparisons.

We subsequently surveyed the frequency of multiple SNPs in each one of these six sequence-based nuclear DNA markers. DNA sequence analyses from distinct *R. solani* AG-1 IA isolates revealed variable levels of polymorphism among markers (Table 3). We also detected variable DNA base ambiguities, typical of heterokaryons, which were efficiently separated using the strategy of cloning and sequencing fragments amplified by PCR.

In comparison to a prior multilocus genotyping system using ten microsatellite loci (Zala *et al.*, 2007), the new set of sequence-based nuclear DNA markers displayed best power for allele discrimination in *R. solani* AG-1 IA. The microsatellite genotyping system indicated the occurrence of four to 10 alleles per locus in 232 soybean-infecting isolates (Ciampi *et al.*, 2008), while up to 18 alleles were identified using our sequence-based markers in a considerably smaller sample of 16 isolates used in this study. These six new sequence-based loci could then be employed as a source of codominant and highly polymorphic SNP markers useful to investigate further questions on the population structure of this important plant pathogen. The chances of finding multiple SNPs are usually highest in non-coding and intergenic regions of the genome, because they are expected to be under less stringent selection than coding regions (van Tienderen *et al.*, 2002). The use of anonymous loci allows markers to be selected without reference to their polymorphism, a feature that some workers consider essential for providing an unbiased description of genomic variation (Brumfield *et al.*, 2003). Loci are often chosen by virtue of their polymorphism content, in part because higher polymorphism implies greater power for inferring population parameters (Epperson, 2005). SNPs might rapidly become the marker of choice for many applications in population ecology, evolution and conservation genetics, because of the potential for higher genotyping efficiency, data quality, genome-wide coverage and analytical simplicity (*e.g.* in modeling mutational dynamics) (Morin *et al.*, 2004). Furthermore, SNPs evolve in a well-described manner for simple mutational models, such as infinite allele sites model (Kimura and Crow, 1964).

Despite the particular importance of SNPs as population genetic markers, our main goal with this research was to develop a set of sequence-based markers that could be useful and informative for studying the phylogeography of *R. solani* AG-1 IA, such as several recent studies that have successfully utilized anonymous regions to infer phylogeographic history (Dettman *et al.*, 2003; Carstens and Knowles, 2007; Ceresini *et al.*, 2007). Up to now only very few sequence-based markers were available for such purposes: ribosomal DNA genes and intergenic regions [such as the ITS-rDNA, commonly used for phylogenetics (Gonzalez *et al.*, 2001; Fenille *et al.*, 2003) and evolutive analyses (Ciampi *et al.*, 2005)], and beta-tubulin gene (Gonzalez *et al.*, 2006). Only recently, two anonymous sequence-based nuclear DNA loci were developed from former PCR-RFLP markers (pP42F e pP89) and used for phylogeography study of the Solanaceae-infecting *R. solani* AG-3 (Ceresini *et al.*, 2007). Large-scale SNP surveys have shown considerable promise for revealing fine-scale population history, assisted by new sequencing technologies that will certainly make these markers a more viable option for studies of natural populations (Brito and Edwards, 2008).

To illustrate the application of the new markers for phylogeographical studies, we performed nested clade analysis (NCA) for locus R44L on haplotypes network of *R. solani* AG-1 IA isolates, constructed using the statistical parsimony algorithm (Templeton *et al.*, 1992) implemented by TCS (Clement *et al.*, 2000) and presented in Figure 1. This network was submitted to a nested design, following rules by Templeton (1987), and tested for geographical association of haplotypes implemented by GeoDis (Posada *et al.*, 2000). It evidences a clade definition by sample origin and/or host: clade 2-1 groups only haplotypes of soybean-infecting isolates from either Mato Grosso or Maranhão State; clade 2-2 groups soybean-infecting haplotypes from Mato Grosso State; and clade 2-3 groups rice-infecting haplotypes from Tocantins State (Figure 1). Based on NCA, a contiguous range expansion was suggested for geographical association of clades, which is coherent with historical processes of dissemination of the pathogen following the expansion of rice and soybean crop areas.

**Figure 1:**
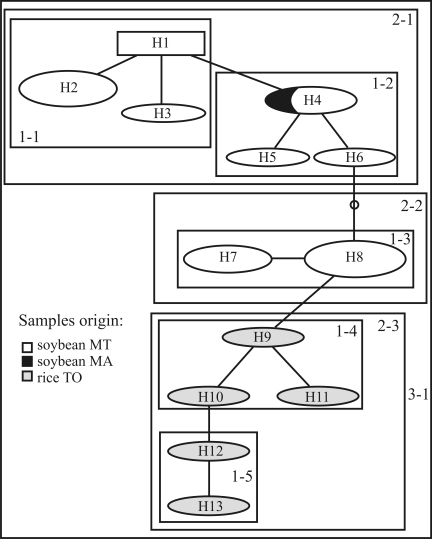
Haplotype network of *Rhizoctonia solani* AG-1 IA for locus R44L, constructed using the statistical parsimony algorithm (Templeton *et al.*, 1992) implemented by TCS (Clement *et al.*, 2000), where haplotypes (H1-H13) form groups represented by circles; the area of each circle refers to the relative frequency of those haplotypes in the population, and the gray tones represent their geographical origin, as shown in the legend. A dot without denomination along the network indicates a putative haplotype not sampled from the population. Probable recombinant haplotypes, identified by sequence homoplasy, were removed from the network. Squares represent the nesting design following the rules proposed by Templeton (1987), which was used to test the geographical association of haplotypes, and was implemented by GeoDis (Posada *et al.*, 2000).

Phylogeographic studies combine information about genetics and population biology, phylogenetics, molecular evolution and historical biogeography to characterize the geographic distribution of pathogen genealogical lineages in the geographic space (referred to as phylogeographic patterns), inferring biogeographic, demographic, and evolutionary process that have shaped these current patterns (Avise, 2000; Knowles and Maddison, 2002; Knowles, 2004). To construct a robust phylogeographic history based on genealogical data, genomic DNA sequences from several independent loci are needed (Knowles, 2004), considering that each DNA sequence has its own genealogy, and that the evolutionary history of an organism is the sum of multiples different gene genealogies, composing a mosaic of genealogic patterns in response to ambient (Hare, 2001; Emerson and Hewitt, 2005). We postulate that the six distinctively variable anonymous DNA regions developed in our study contain multiple and linked single nucleotide polymorphisms (SNPs) essential for constructing and comparing multi-locus gene genealogies required in any phylogeography study. Phylogeographic studies using genealogical data from these independent loci would provide a more reliable species history containing the phylogeographic patterns that shaped the current population structure of *R. solani* AG-1 IA.

## Supplementary Material

The following online material is available for this article:

Table S1 Detailed description of molecular variation within six nuclear DNA sequence-based markers from *Rhizoctonia solani* AG-1 IA isolates.

This material is available as part of the online article from http://www.scielo.br/gmb.

## Figures and Tables

**Table 1:** *Rhizoctonia solani* AG-1 IA isolates used in this study.

Isolate	Host	Source	Origin	ITS haplotype^1^	GenBank accession number
3F1	rice cv. Epagri 108	A.S. Prabhu	Lagoa da Confusão, TO	5	DQ173049.1
3F6	rice cv. Rio Formoso	”	”	5	DQ173050.1
4F1	rice cv. Epagri 108	”	”	5	DQ173051.1
9F1	”	”	”	5	DQ173052.1
SJ13	soybean cv. Garça Branca	R.C. Fenille	Lucas do Rio Verde, MT	22	DQ173053.1
SJ15	”	”	”	20	DQ173055.1
SJ16	”	”	”	14	AY270010.1
SJ19	”	”	”	12	AY270013.1
SJ28	soybean cv. Xingu	”	”	23	AY270006.1
SJ31	”	”	”	1	DQ173058.1
SJ34	soybean cv. FT-108	”	”	19	AY270007.1
SJ36	”	”	”	13	DQ173060.1
SJ40	”	”	”	10	DQ173061.1
SJ44	”	”	”	2	DQ173062.1
SJ47	”	”	”	9	DQ173063.1
SJ53	”	”	”	17	DQ173065.1
SJ93	soybean	M.C. Meyer	Pedro Afonso, TO	18	DQ173068.1
SJ129	soybean cv. Sambaiba	”	Balsas, MA	16	DQ173071.1

^1^ITS-5.8S haplotypes characterized by Ciampi *et al.* (2005).

**Table 2:** Characteristics of ten nuclear DNA sequencing markers developed for *Rhizoctonia solani* AG-1 IA.

Locus	Product size (bp)	Primer pair	Primer sequence (5' - 3')	Tm	GC%
R44L	303	R44LL	AGACGTACTCTGTCCAGACCAA	58.9	50.0
		R44LR	GAATAGGTTTCTGCCCTCTTCG	61.4	50.0

R61L	281	R61LL	GGACCTTGGCTTAGGAAAGAAG	60.6	50.0
		R61LR	AGTGACGCTTGCTCAGACTAGG	61.1	54.6
R61R	300	R61RL	ATCGCAAGAAACCAGACTGC	60.4	50.0
		R61RR	CGAATATCGCCCATCGTACT	59.9	50.0

R68L	303	R68LL	AGACTGTTGACTGGTGTGATCG	60.2	50.0
		R68LR	CAGCGCTGCGTACTACAGCTA	61.8	57.1

R78L	195	R78LL	ATATGGCACCTGACCTCGAC	60	55.0
		R78LR	CGAGTTTGCCCATACTTGGT	60	50.0

R111R	241	R111RL	GTGAGCGCCAGACAAGAGATA	60.6	52.4
		R111RR	ATTCCCAAGTCAGCAGCAGT	59.9	50.0

R116L	314	R116LL	CACAGATCCAGAGGTTGTGC	59.3	55.0
		R116LR	TGCTTCCAGCGTACATTCTG	60	50.0
R116R	223	R116RL	CGTTAGTATCGAGGTAGCCACA	59.3	50.0
		R116RR	GACCGTAGACAGGAGAAGATCG	60.3	54.6

R148L	320	R148LL	CCGTCCGTTATCCGACTTACTA	60.3	50.0
		R148LR	CCGTCCGTTATCCGACTTACTA	60.4	50.0
R148R	201	R148RL	AGCAGCATGCCGAGTTGATA	61.9	50.0
		R148RR	GTCGGTATGTCACAGACGAATG	60.4	50.0

**Table 3:** Descriptive analysis of molecular variation within six nuclear DNA sequencing markers from *Rhizoctonia solani* AG-1 IA isolates.

Locus	Product size (bp)	Number of isolates surveyed	Number and proportion of heterokaryotic isolates	Number of sequences analyzed^1^	Number of haplotypes detected	Number of polymorphic sites	Indels	Number of mutations/100 bp	Hd^2^	π^3^	k^4^	NCBI-GenBank accession number
R44L	303	16	14 = 0.88	30	17	10	0	3.3	0,938 ± 0,025	0.011	3.267	EU907373-EU907402
R61R	300	16	5 = 0.31	21	10	10	1	3.3	0,900 ± 0,039	0.010	2.848	EU907408-EU907428
R68L	303	16	4 = 0.25	21	11	18	0	5.9	0,857 ± 0,057	0.025	7.686	EU907471-EU907491
R116L	313	15	3 = 0.20	18	12	13	2	4.2	0,922 ± 0,047	0.013	4.176	EU907435-EU907452
R148L	320	4	3 = 0.75	7	5	8	2	2.5	0,857 ± 0,137	0.008	2.667	EU907453-EU907459
R148R	200	11	0	11	2	1	2	0.5	0,545 ± 0,072	0.003	0.545	EU907460-EU907470

^1^The total number of sequences analyzed is higher than the number of isolates surveyed because most of the individuals were heterokaryons, requiring proper separation of alleles from each heterogeneous sequence by cloning. ^2^Haplotype diversity (Hd) ± standard deviation, calculated according to Nei (1987). ^3^Nucleotide diversity (π) or average number of differences per site between two sequences, calculated according to Nei (1987), Eq. (10.5). π values were estimated as the average among all comparisons, for each marker. ^4^The average number of nucleotide differences (Tajima 1983, Eq. (A3)).
