# Supplementary material for: Distinctively variable sequence-based nuclear DNA markers for multilocus phylogeography of the soybean- and rice-infecting fungal pathogen Rhizoctonia solani AG-1 IA
Source: Genet Mol Biol. 2009 Dec 1;32(4):840–6. doi: 10.1590/S1415-47572009005000063 (PMC3036909; doi:10.1590/S1415-47572009005000063)
Supplement: Table S1 — Detailed description of molecular variation within six nuclear DNA sequence-based markers from Rhizoctonia solani AG-1 IA isolates. [file gmb-32-4-840-suppl1.pdf]

Table S1 - Detailed description of molecular variation within six nuclear DNA sequence-based markers from *Rhizoctonia solani* AG-1 IA isolates.

Locus	Haplotype number	Haplotype frequency	Polymorphic position along DNA sequence										Isolates sharing the haplotype[1]
			31	37	64	74	95	100	119	161	269	292	
	H1	1	T	C	T	G	T	A	A	T	T	G	SJ13R44*a*
R44L	H2	1	C	C	T	G	T	A	G	T	T	G	SJ36R44*b*
	H3	1	C	T	C	A	T	G	G	C	T	G	3F6R44*b*
	H4	5	C	T	C	G	T	A	G	T	T	G	SJ16R44*b*, SJ34R44*b*, SJ40R44*b*, SJ47R44*b*, SJ53R44*b*
	H5	3	C	T	C	G	T	A	G	T	C	G	SJ19R44*b*, SJ28R44*b*, SJ31R44*b*
	H6	1	C	T	C	A	T	A	G	C	T	G	9F1R44*b*
	H7	1	C	T	C	A	T	A	G	T	T	G	3F6R44*a*
	H8	1	C	T	C	A	T	A	G	T	T	A	4F1R44*a*
	H9	1	C	T	C	A	T	G	G	T	T	G	9F1R44*a*
	H10	1	C	T	C	A	T	G	G	C	T	A	4F1R44*b*
	H11	1	T	T	C	A	T	A	G	T	T	G	SJ36R44*a*
	H12	1	T	C	T	A	T	A	G	T	C	G	SJ16R44*a*
	H13	5	T	C	T	A	T	A	G	T	T	A	SJ28R44*a*, SJ31R44*a*, SJ34R44*a*, SJ40R44*a*, SJ53R44*a*
	H14	2	T	C	T	A	T	A	G	T	T	G	SJ19R44*a*, SJ47R44*a*
	H15	1	T	C	T	G	C	A	G	T	T	A	SJ93R44*b*
	H16	3	T	C	T	G	T	A	G	T	T	G	SJ129R44, SJ15R44, SJ13R44*b*
	H17	1	T	C	T	G	T	A	G	T	C	G	SJ93R44*a*
	N[2] =	30											
			85	133	136	169	232	244	292	294	295	296	
	H1	2	A	T	A	A	C	A	G	G	C	G	3F1R61R, 3F6R61R
R61R	H2	1	G	T	A	A	C	A	G	G	C	G	SJ15R61R*a*
	H3	5	G	T	A	G	C	A	G	G	C	G	SJ93R61R, SJ129R61R*b*, SJ40R61R, SJ36R61R*a*, SJ34R61R
	H4	1	G	T	A	G	C	G	G	G	C	G	SJ129R61Ra
	H5	3	G	T	A	G	C	A	A	G	C	G	SJ13R61R*b*, SJ47R61R, SJ53R61R
	H6	1	G	T	A	A	T	A	G	C	G	A	9F1R61R*b*
	H7	4	G	T	A	A	C	G	G	G	C	G	SJ15R61R*b*, SJ28R61R, SJ31R61R, SJ36R61R*b*
	H8	1	G	T	A	A	C	G	A	G	C	G	SJ13R61R*a*
	H9	2	G	T	A	A	C	G	G	C	G	A	SJ16R61R, SJ19R61R
	H10	1	A	C	G	A	C	A	G	C	G	A	9F1R61R*a*
	N[2] =	21											

Locus	Haplotype number	Haplotype frequency	Polymorphic position along DNA sequence																		Isolates sharing the haplotype[1]
			35	53	74	96	126	132	135	160	179	186	193	206	209	216	242	264	272	276	
	H1	1	G	T	T	A	C	G	A	C	G	T	G	G	T	A	G	A	A	A	3F1R68L*b*
R68L	H2	1	T	T	T	A	C	G	A	C	G	T	C	A	T	A	G	A	G	A	3F6R68L*b*
	H3	1	T	T	C	C	T	A	G	T	A	T	G	G	T	A	A	G	A	A	3F6R68L*a*

	H4	6	T	T	C	A	C	A	A	C	G	T	G	G	T	A	A	G	A	A	SJ93R68L, SJ129R68L, SJ31R68L, SJ28R68L, SJ16R68L, SJ13R68L
	H5	1	T	T	C	A	C	A	G	C	G	C	G	G	T	A	A	G	A	A	4F1R68L*a*
	H6	1	T	T	C	A	C	A	A	C	G	T	G	G	T	A	A	G	A	G	SJ19R68L
	H7	1	T	T	T	A	T	A	G	T	A	T	G	A	T	G	A	A	A	A	3F1R68L*a*
	H8	1	T	T	T	A	T	A	G	C	G	T	G	G	T	A	A	G	A	A	9F1R68L*a*
	H9	1	G	G	T	A	C	G	A	C	A	T	G	G	C	A	A	G	A	A	4F1R68L*b*
	H10	6	G	G	T	C	T	G	G	C	A	C	C	A	C	A	A	G	G	G	SJ34R68L, SJ36R68L, SJ40R68L, SJ44R68L, SJ47R68L, SJ53R68L
	H11	1	G	G	T	A	C	A	A	C	A	C	G	A	C	A	A	G	A	A	9F1R68L*b*
	N[2] =	21																			
			36	41	46	51	62	68	70	106	121	125	144	157	198						
	H1	1	A	G	A	G	C	C	A	G	A	C	C	A	G						3F1R116*a*
R116L	H2	1	G	C	A	A	C	C	A	G	A	C	T	A	G						SJ36R116
	H3	4	A	C	A	A	C	C	A	G	A	C	T	A	G						SJ53R116, SJ47R116, SJ44R116, SJ40R116
	H4	1	A	C	T	A	C	C	A	G	A	C	T	A	G						SJ129R116*b*
	H5	1	A	C	A	G	C	C	A	G	A	C	T	A	G						SJ93R116*b*
	H6	4	A	C	A	G	A	C	T	T	A	C	C	G	A						SJ16R116, SJ19R116, SJ28R116, SJ31R116
	H7	1	A	C	A	G	C	C	A	G	A	C	C	A	G						3F6R116
	H8	1	A	C	A	A	A	C	A	G	A	C	T	A	G						SJ93R116*a*
	H9	1	A	C	A	A	A	T	A	G	T	C	T	A	G						SJ129R116*a*
	H10	1	A	G	A	A	C	C	A	G	A	C	T	A	G						SJ34R116
	H11	1	A	G	A	G	A	C	T	T	A	C	C	G	A						4F1R116
	H12	1	A	G	A	G	C	C	A	G	A	T	C	A	G						3F1R116*b*
	N[2] =	18																			
			123	126	170	200	221	223	253	266											
	H1	1	C	T	G	T	A	A	A	T											SJ13.R148L*a*
R148L	H2	1	G	G	G	T	A	G	G	C											SJ93.R148L*b*
	H3	1	C	T	C	T	A	G	A	T											SJ36.R148L
	H4	1	C	T	G	G	T	G	G	C											SJ129.R148L*b*
	H5	3	C	T	G	T	A	G	A	T											SJ13.R148L*b*, SJ93.R148L*a*, SJ129.R148L*a*
	N[2] =	7																			
Locus	Haplotype number	Haplotype frequency	Polymorphic position along DNA sequence																		Isolates sharing the haplotype[1]
			68																		
R148R	H1	6	T																		SJ36.R148R, SJ53.R148R, SJ16.R148R, SJ19.R148R, SJ28.R148R, SJ31.R148R
	H2	5	C																		3F1.R148R, 3F6.R148R, 4F1.R148R, 9F1.R148R, SJ15.R148R
	N[2] =	11																			

[1]Heterokaryotic isolates identified by two sequences (*a* and *b*), exhibiting distinct alleles.
[2]Total numbers of sequences analyzed. Includes a single sequence for every homokaryotic isolates and two sequences for each heterokaryotic one (as a result of ambiguous DNA base separation by cloning PCR products of each nuclear marker).
